# Retrograde Transvenous Lymphatic Embolization for Refractory Chylous Ascites after Laparoscopic Distal Gastrectomy: A Case Report

**DOI:** 10.70352/scrj.cr.26-0495

**Published:** 2026-10-02

**Authors:** Sangyo Kashima, Shuntaro Yoshimura, Joe Toda, Kazuhiko Mori

**Affiliations:** Department of Digestive Surgery, Mitsui Memorial Hospital, Tokyo, Japan

**Keywords:** retrograde transvenous lymphangiography, lymphatic embolization, postoperative, chylous ascites, laparoscopic gastrectomy, gastric cancer, case report

## Abstract

**INTRODUCTION:**

Chylous ascites is a rare complication of gastric cancer surgery and is usually managed conservatively. However, treating refractory cases with persistent high-output drainage remains challenging. Although lymphangiography with subsequent embolization has recently emerged as a minimally invasive treatment option, the identification of the leakage site is often difficult using conventional intranodal approaches. In such cases, retrograde transvenous lymphangiography and embolization may be feasible and safe. Herein, we describe a case of refractory postoperative chylous ascites after laparoscopic gastrectomy that was successfully treated with retrograde transvenous lymphangiography and subsequent embolization.

**CASE PRESENTATION:**

An 85-year-old man underwent a laparoscopic distal gastrectomy with D1+ lymph node dissection for gastric cancer. On POD 4, chylous ascites developed after the initiation of oral intake. Despite conservative management, including fasting and the administration of a somatostatin analog, the high-output drainage persisted at 1000–2000 mL/day, resulting in severe hypoalbuminemia. Intranodal lymphangiography failed to identify the leakage site. Therefore, retrograde transvenous lymphangiography was performed through the left venous angle, which revealed leakage at the level of the 3rd lumbar vertebra. Subsequently, lymphatic embolization was performed, which led to a rapid decrease in the drainage output. No complications were observed during the procedure. The patient recovered without any recurrence and was discharged 22 days after the procedure.

**CONCLUSIONS:**

Retrograde transvenous lymphatic embolization may be an effective and safe treatment option for refractory chylous ascites after gastrectomy, particularly when the leakage site cannot be identified using conventional intranodal lymphangiography.

## INTRODUCTION

Chylous ascites is a rare complication of gastric cancer surgery, with an incidence of less than 1%.^1)^ It is generally caused by injury to major lymphatic channels, such as the cisterna chyli or the lymphatic vessels around the celiac axis, during extragastric lymph node dissection. Most cases occur in the early postoperative period and are diagnosed on the basis of milky or creamy fluid drainage with elevated triglyceride levels in the ascites.^2)^ Conservative management, including dietary fat restriction, total parenteral nutrition, and the administration of somatostatin analogs, is considered as first-line treatment and is effective in most patients.^3)^ However, treating refractory cases with persistent high-output drainage remains challenging.

Several treatment options for refractory cases of chylous ascites have been reported, including surgical intervention under general anesthesia, peritoneovenous shunting,^4)^ and interventional radiological procedures.^3)^ Additionally, lymphangiography with subsequent lymphatic embolization has recently emerged as a minimally invasive approach, with intranodal lymphangiography performed in most cases. However, in cases of disrupted lymphatic flow after extensive lymph node dissection, identification of the leakage site by intranodal lymphangiography can be challenging. Indeed, previous reports indicate that the leakage site cannot be identified in approximately half of cases treated using intranodal lymphangiography.^5,6)^ We considered a retrograde transvenous approach through the left venous angle to be potentially useful in such cases.

Here, we report a case of refractory chylous ascites after laparoscopic distal gastrectomy for gastric cancer, in which the leakage site was successfully identified and treated using retrograde transvenous lymphangiography and subsequent embolization.

## CASE PRESENTATION

An 85-year-old man underwent periodic endoscopic upper gastrointestinal surveillance for atrophic gastritis at another hospital. He was 155 cm tall and weighed 53.2 kg (BMI, 22.0 kg/m^2^). His medical history included aortic valve replacement for aortic stenosis, surgery for lumbar spinal stenosis, and appendectomy for appendicitis. He had no notable medical history considered to increase the risk of postoperative lymphatic leakage.

Two months prior to admission, upper gastrointestinal endoscopy revealed a 30-mm, type-2 lesion located on the lesser curvature of the gastric angle (**Fig. 1A** and **1B**). Histopathological examination of biopsy specimens revealed moderately to poorly differentiated adenocarcinoma. No lymph node metastasis was observed on CT. The preoperative clinical stage was clinical T2N0M0 Stage I.

**Fig. 1:**
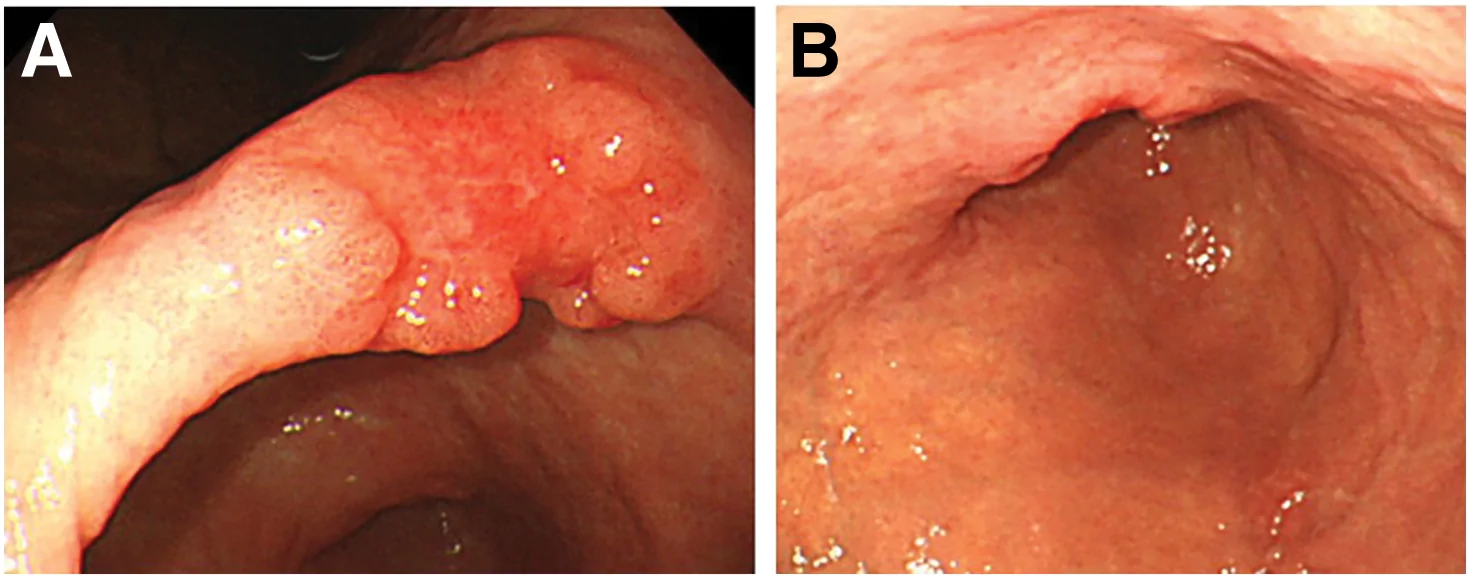
Endoscopic findings. (**A**, **B**) Upper gastrointestinal endoscopy reveals a 30-mm type-2 lesion located on the lesser curvature of the gastric angle.

Laparoscopic distal gastrectomy with extragastric lymph node (D1+) dissection was performed. An ultrasonic coagulating shear device, the HARMONIC 1100 Shears (Ethicon, Cincinnati, OH, USA), was used during the procedure. During suprapancreatic lymph node dissection, the bottom of lymph nodes 8a and 9 were dissected using the ultrasonic coagulating shear device without clip application. Reconstruction was performed using the Roux-en-Y method, and a drain was placed along the superior border of the pancreas from the right upper quadrant. The operative time was 270 minutes, and the estimated blood loss was 40 mL.

On POD 4, following the initiation of oral intake, the drainage volume increased and the fluid became milky in appearance, suggesting chylous ascites. The triglyceride concentration of the drainage fluid measured on POD 5 was 234 mg/dL. Conservative management was initiated, including fasting (water intake was permitted), administration of a somatostatin analog and diuretics, and total parenteral nutrition with ELNEOPA (Otsuka Pharmaceutical, Tokyo, Japan; initiated with No. 1 solution and switched to No. 2 solution after 2 days, 2000 mL/day, approximately 1120 and 1640 kcal/day, respectively) supplemented with intravenous lipid emulsion with Intralipos 20% (Otsuka Pharmaceutical; 100 mL/day, 20 g fat, 200 kcal/day). On POD 10, oral intake was resumed with a fat-restricted diet; however, because the drainage output remained approximately 1000 mL/day, fasting and total parenteral nutrition were reinstituted on POD 15 in an attempt to achieve resolution with continued conservative treatment. Despite these measures, the drainage volume progressively increased to approximately 1500–2000 mL/day by POD 18, resulting in dehydration and severe hypoalbuminemia (lowest serum albumin level, 1.7 g/dL), which required repeated intravenous albumin administration. As conservative treatment was considered unsuccessful and difficult to continue because of persistent high-output chylous ascites and progressive nutritional deterioration, interventional radiology was planned.

On POD 23, intranodal lymphangiography via the inguinal lymph node was performed to identify the site of leakage. Under US guidance, the right inguinal lymph node was punctured using a 23-gauge needle, and lipiodol was slowly injected. However, no obvious cisterna chyli was observed, and the leakage site could not be detected (**Fig. 2**). The contrast agent flowed rapidly through the thoracic duct toward the left venous angle.

**Fig. 2:**
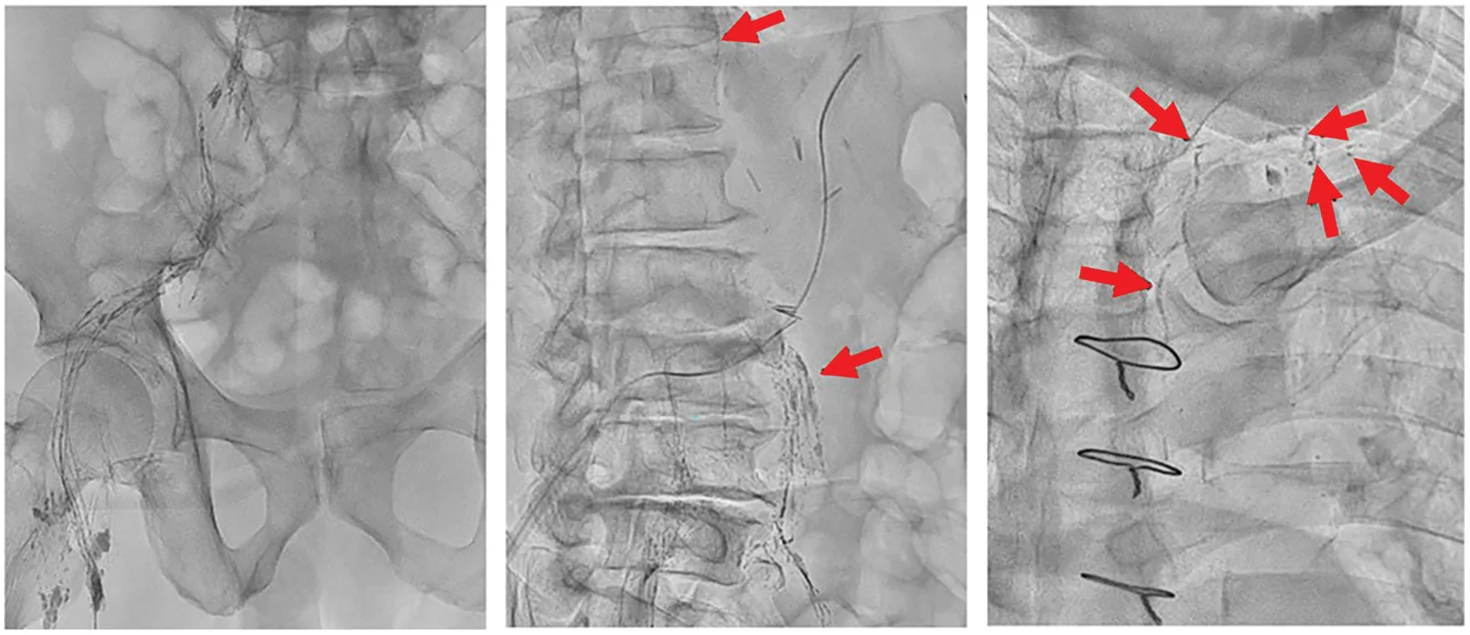
Intranodal lymphangiography. Lymphangiography via the right inguinal lymph node fails to reveal the leakage site. The contrast medium can be seen rapidly flowing into the thoracic duct toward the left venous angle (red arrows).

Therefore, on POD 25, retrograde lymphangiography was performed through the left venous angle. Under US guidance, a 5-Fr, 25-cm sheath was inserted into the left brachial vein. A microcatheter (0.016-inch; ASAHI Meister, Asahi Intecc, Aichi, Japan) was advanced into the thoracic duct via the left venous angle and then into the abdominal lymphatic system (**Fig. 3A**). Contrast injection revealed leakage from the lymphatic vessels on the right side of the 3rd lumbar vertebra (L3) level (**Fig. 3B**). Subsequently, lymphatic embolization was performed. Microcoils (0.018-inch, 2 mm × 6 cm and 2 mm × 4 cm; Interlock, Boston-Scientific, Marlborough, MA, USA) were deployed at the site of leakage and a 1:1 mixture of N-butyl cyanoacrylate and lipiodol (total volume, 0.5 mL) was injected through the microcatheter (**Fig. 3C**). A CT scan performed immediately after embolization demonstrated contrast extravasation on the right side of the L3 and pooling between the dorsal aspect of the inferior vena cava and the right posterior pararenal space (**Fig. 4A** and **4B**). No procedure-related complications were observed.

**Fig. 3:**
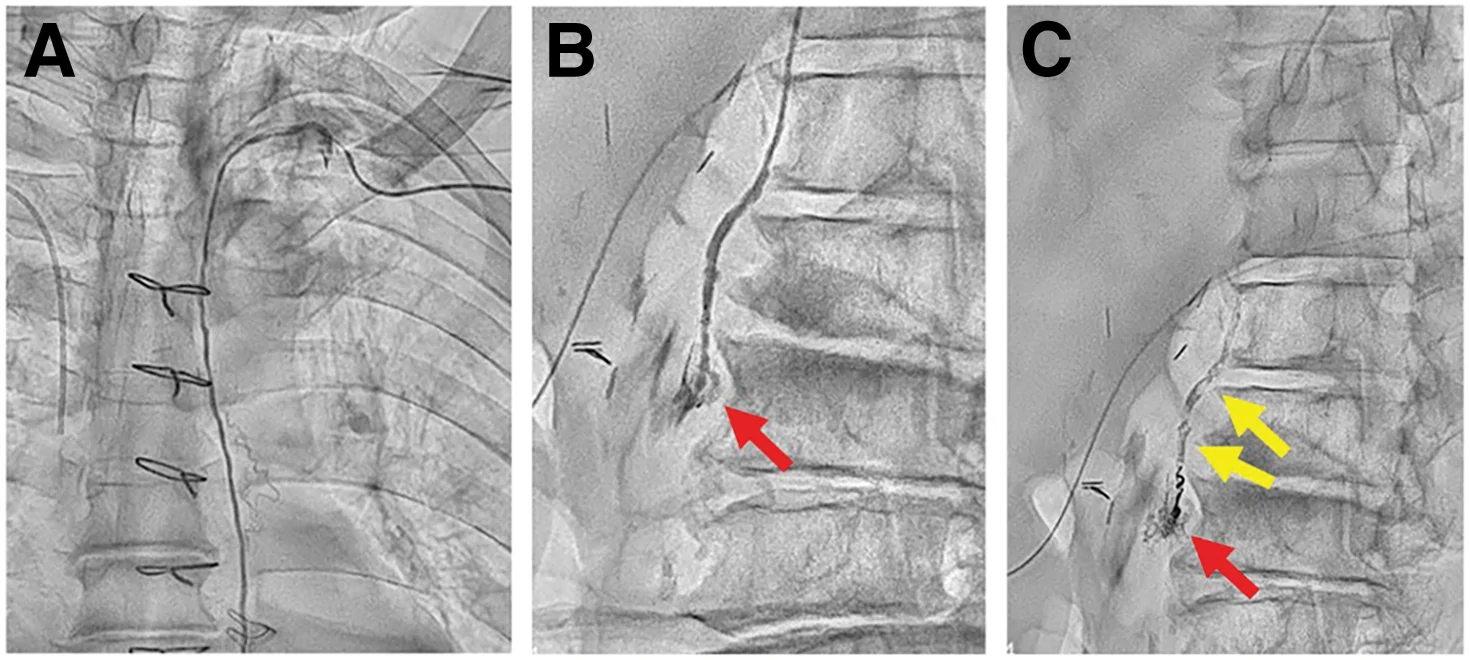
Retrograde transvenous lymphangiography and embolization. (**A**) A microcatheter is advanced into the thoracic duct via the left venous angle. (**B**) Contrast injection reveals lymphatic leakage on the right side at the level of the L3 (red arrow). (**C**) Embolization is performed using microcoils (red arrow), followed by injection of a mixture of N-butyl cyanoacrylate and lipiodol (yellow arrows). L3, 3rd lumbar vertebra

**Fig. 4:**
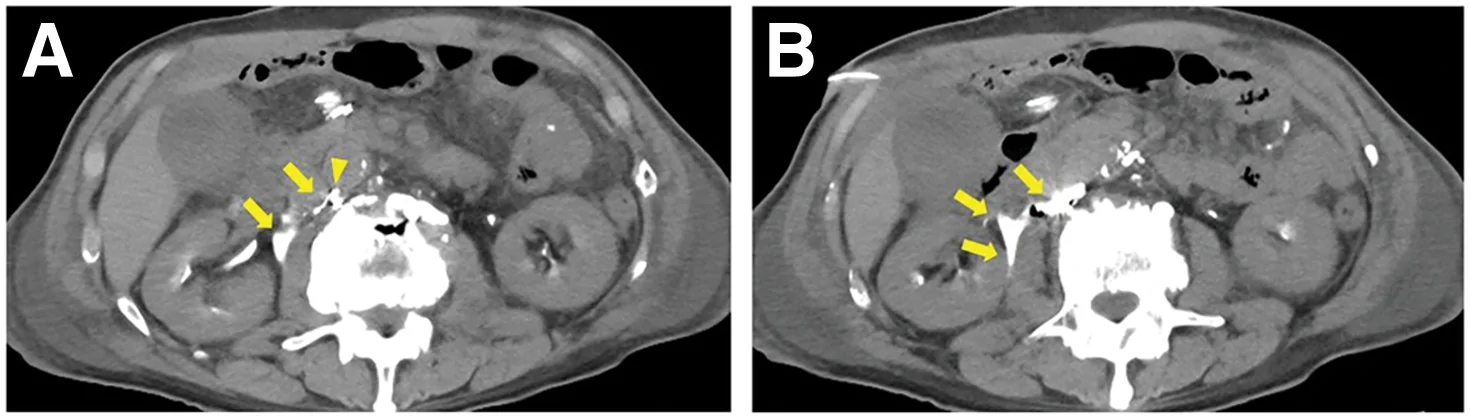
CT findings after embolization. (**A**, **B**) Contrast extravasation is observed on the right side of the L3 level, with pooling between the dorsal aspect of the inferior vena cava and the right posterior pararenal space (yellow arrows). L3, 3rd lumbar vertebra

After embolization, the drainage output decreased rapidly, and no increase was observed after the resumption of oral intake (**Fig. 5**). The drain was removed 16 days after embolization. The patient experienced no recurrence of chylous ascites and was discharged 22 days after embolization.

**Fig. 5:**
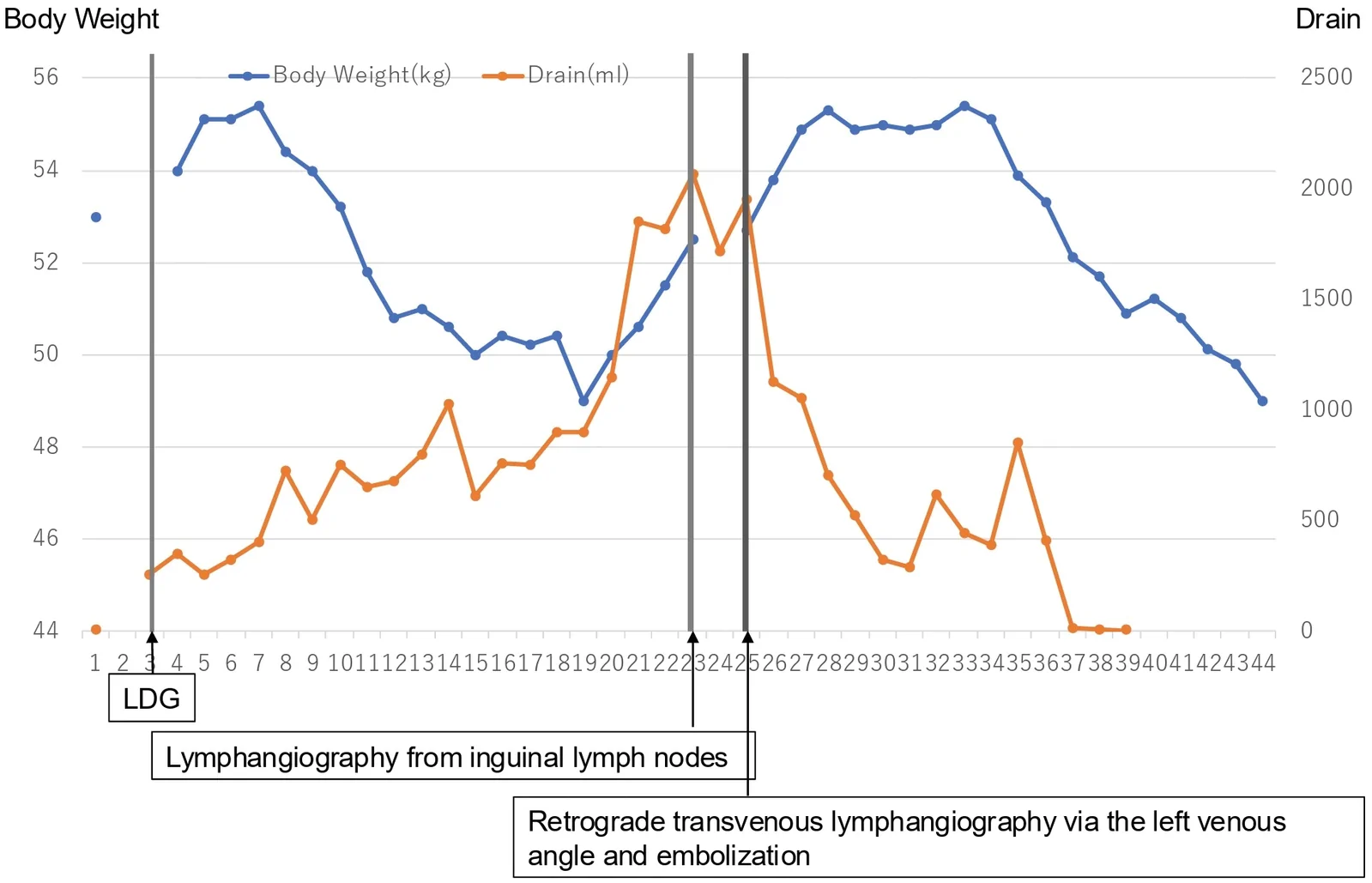
Clinical course showing a rapid decrease in drainage output after lymphatic embolization. LDG, laparoscopic distal gastrectomy

The pathological diagnoses were M, Less, 0–IIc, 24 × 15 mm, pathological T1b2 (SM2), INFc, Ly1a, V0, pPM0, pDM0, N0 (0/15), and pathological Stage IA. No tumor recurrence has been observed for more than 3 years.

## DISCUSSION

In the present case, refractory postoperative chylous ascites was successfully diagnosed and treated by retrograde transvenous lymphangiography and embolization without any complications. This suggests that retrograde transvenous lymphatic embolization can serve as an effective and safe minimally invasive alternative when intranodal lymphangiography fails to identify the leakage site, potentially avoiding the need for reoperation.

Chylous ascites after gastric cancer surgery is a rare complication, and its reported incidence after laparoscopic gastrectomy for gastric cancer ranges from approximately 0.3% to 0.7%.^1)^ Chylous ascites after abdominal surgery is generally considered to result from direct injury to the lymphatic system during lymph node dissection or from lymphatic obstruction,^7)^ and, in particular, inadequate sealing of the lymphatic vessels during the dissection of lymph nodes 8a and 9 may contribute to its development after gastric cancer surgery.^8)^ Consistent with this mechanism, chylous drainage often becomes clinically evident only after the resumption of oral intake or an increase in dietary fat intake, presumably reflecting increased lymphatic flow through the injured vessels.^9)^ In the present case, suprapancreatic lymph node dissection was performed using an ultrasonic coagulating shear device, and the identified leakage site was consistent with the area of suprapancreatic dissection, supporting direct injury of the lymphatic vessel as the likely cause.

Postoperative chylous ascites is typically defined as the appearance of milky and non-purulent drainage fluid with a triglyceride concentration exceeding 200 mg/dL.^2)^ Conservative management is considered as the first-line treatment and includes fat-restricted diets or fasting, total parenteral nutrition and drainage, and, in some cases, somatostatin analog administration.^3)^ Although conservative therapy is effective in approximately 70%–80% of cases, persistent chylous ascites may lead to severe nutritional deterioration, as in the present case. Lv et al. reported that surgical intervention is generally considered when conservative management fails, chylous leakage persists for more than 2 weeks, high-output drainage exceeding 1000 mL/day continues for more than 1 week, or nutritional deterioration develops.^3)^ In the present case, oral intake with a fat-restricted diet was attempted from POD 10, but the drainage output remained at approximately 1000 mL/day. By POD 18, the output had increased to 1500–2000 mL/day, accompanied by severe hypoalbuminemia, thereby fulfilling the criteria for considering intervention. The marked increase in drainage fluid observed around POD 18 may not have reflected worsening lymphatic leakage alone. The serum albumin level had declined to the lowest level of 1.7 g/dL, while the ascitic fluid albumin concentration was 1.2 g/dL, suggesting that severe hypoalbuminemia may have contributed to fluid transudation into the peritoneal cavity.

Reoperation is highly invasive, and lymphangiography with or without subsequent embolization has emerged as a less invasive alternative.^5)^

Antegrade intranodal lymphangiography is a technique in which a contrast material, such as lipiodol, is injected into an inguinal lymph node under US guidance, followed by CT to visualize the lymphatic system and identify the site of lymphatic leakage. Although lymphangiography has been performed using the pedal approach, in recent years, the intranodal approach has been mainly used.^10)^ In addition to its diagnostic role, lymphangiography itself can have a therapeutic effect, possibly due to the inflammatory reaction and subsequent occlusion of lymphatic vessels caused by the contrast medium.^6)^ However, lymphangiography via the intranodal approach is often difficult to perform after extensive lymph node dissection, as the disruption of normal lymphatic flow can prevent adequate visualization of the lymphatic system.^11)^ In such cases, a retrograde transvenous approach may enable visualization of the lymphatic system and facilitate subsequent embolization.^11,12)^

In contrast to antegrade lymphangiographic techniques, such as intranodal or pedal lymphangiography, retrograde transvenous lymphangiography involves the injection of contrast medium into the basilic or cephalic vein retrogradely through the thoracic duct via the left venous angle to visualize the abdominal lymphatic system. This approach may be effective when the site of injury is located upstream of the cisterna chyli, as in the present case.^11)^

However, this technique has several limitations, including technical difficulties due to anatomical variations. Challenges to retrograde access include a thick and short neck, tortuous or plexiform termination of the thoracic duct, and potential anatomical anomalies throughout the course of the thoracic duct or at its termination. In addition, advancement of the catheter to the upstream thoracic duct or cisterna chyli can be difficult because of the presence of valves and anatomical anomalies of the thoracic duct, such as duplication or plexiform variations.^13)^ A previous report demonstrated a technical success rate of 61.5% (8 of 13 patients) for retrograde transvenous lymphangiography performed in a mixed population with chylous leakage or lymphatic complications following various surgical and nonsurgical procedures. Among these 13 patients, 2 had chylous ascites after gastric cancer surgery, of whom 1 was successfully treated with this technique.^13)^

To the best of our knowledge, this is one of the few reported cases of refractory chylous ascites after gastric cancer surgery in which the leakage site was successfully identified and treated with retrograde transvenous lymphatic embolization via the left venous angle. Although similar approaches have been reported in patients who underwent esophagectomy, nephrectomy, or gynecological surgery, this technique may also be useful in the management of chylous ascites following gastric cancer surgery.^11,14–17)^ Notably, although Kariya et al. previously reported successful treatment in 1 of 2 patients with chylous ascites after gastric cancer surgery, the surgical approach in that case was not specified.^14)^ Our experience suggests that this approach is both feasible and safe, particularly when intranodal lymphangiography fails to identify the site of lymphatic leakage.

## CONCLUSIONS

We described a case of refractory chylous ascites after laparoscopic gastric cancer surgery that was successfully treated by retrograde transvenous lymphatic embolization. No complications were observed during the procedure. Thus, retrograde transvenous lymphatic embolization may be an effective and safe treatment option for postoperative chylous ascites and should be considered before surgical intervention.

## DECLARATIONS

### Availability of data and materials

All data generated or analyzed during this study are included in this published article.
